# Structural and Optical Characteristics of Highly UV-Blue Luminescent ZnNiO Nanoparticles Prepared by Sol–Gel Method

**DOI:** 10.3390/ma13040879

**Published:** 2020-02-15

**Authors:** Ashraf H. Farha, Abdullah F. Al Naim, Javed Mazher, Olfa Nasr, Mohamed Helmi Hadj Alouane

**Affiliations:** 1 Department of Physics, College of Science, King Faisal University, P.O. Box: 400, Al-Ahsa 31982, Saudi Arabia; afarha@kfu.edu.sa (A.H.F.); anaim2@kfu.edu.sa (A.F.A.N.); onasr@kfu.edu.sa (O.N.); malouane@kfu.edu.sa (M.H.H.A.); 2 Semiconductors Technology Lab, Physics Department, Faculty of Science, Ain Shams University, 11566 Cairo, Egypt

**Keywords:** nanoparticles, sol-gel, Raman spectroscopy, UV-blue photoluminescence, ZnNiO, Kubelka-Munk function, Burstein-Moss shift, substitutional doping

## Abstract

A simple single pot sol–gel method is used to prepare ZnNiO nanoparticles at assorted Ni doping levels, 1, 3, 7 and 10 wt.%. Structural and optical properties of nanoparticles are studied by X-ray diffraction (XRD), UV–visible diffuse reflection spectroscopy (DRS), photoluminescence (PL) measurements, scanning electron microscopy (SEM), μ-Raman and X-ray photoelectron-spectroscopy (XPS). A single substitutional solid solution phase is detected in the wurtzite ZnNiO nanoparticles at various doping levels. XRD peak splitting and shifting is ascribed to reduced wurtzite character and presence of crystalline strain in nanoparticles at higher level of Ni doping. The Kubelka-Munk function of DRS data reveals the presence of the Burstein-Moss effect in the optical absorption of ZnNiO nanoparticles. Photoluminescence studies show intense UV-blue emission from ZnNiO nanoparticles. The UV PL also exhibits the Burstein-Moss blue shift in the ZnNiO luminescence. Raman analyses also confirms the wurtzite structure of ZnNiO nanoparticles; however, crystal structural defects and bond stiffness increase with Ni doping. The optical and structural studies presented in this work are pointing towards a multivalent Ni substitution in the nanoparticles.

## 1. Introduction:

The presence of intense luminescence in diluted magnetic semiconductor (DMS) materials has been a focus of current research interest owing to potential applications in spin-based all-optical switching, opto-spintronics and opto-spin-based computation [1,2,3,4,5,6]. Fabrication of miniaturized smart devices requires both better optoelectronic and magneto-opto-electronic efficiencies through better incorporation of dopant in nanocrystalline ZnO-based DMS systems [7,8,9,10,11]. The metal (TM) atom incorporation achieved from Ni doping materials in the ZnO lattice has been shown to improvise the integration of both semiconducting and magnetic properties for efficient opto-spintronics applications [12,13,14,15,16]. The success of TM ion doping in ZnO has been evident from observations of room temperature ferromagnetic (FM) ordering in ZnO and Fe atom induced bandgap tuning proved from the diffused reflectance spectroscopy of ZnFeO nanocrystals [17,18]. Moreover, novel phenomena such as poloronic magneto-resistance (MR), superior surface catalysis and super-capacitive behaviors, thermal activation of carriers, and lowering of electrical resistance are also expected in the ZnNiO systems [19,20,21,22].

It is crucial to better understand the mechanisms of incorporation of TM dopant in the host lattice because opto-electronic and magneto-electronic properties significantly depend on the synthesis methods used in preparation of TM doped ZnO nanoparticles [14,18,23]. Jlassi et al. estimated that up to 2% Ni incorporation in ZnO prepared by spray pyrolysis technique can increase n-type carriers through O-vacancy defects [20]. Meanwhile, the free carriers are less likely generated in the sol–gel prepared ZnNiO due to large presence of interstitial Ni ions [24]. However, the single-pot sol–gel synthesis of ZnNiO nanoparticles can give different results due to simultaneous formation of cationic sub-lattice and higher photoluminescence yield can be expected due to presence of higher carrier concentrations [8].

A successful incorporation of Ni dopant in the nanocrystalline ZnO particles can also make them deployable in varisters, as battery electrode, as oxidative catalytic thin-films, super-capacitor electrodes, and so on [25,26,27,28]. Additional synthesis-dependent variables, such as the level of oxygen vacancies and higher dopant-to-dopant near-neighbor interactions, also play a significant role in the enhancement of optoelectronic and opto-spintronic properties of TM doped metal oxides [13,18,29,30,31]. A steep decrease in ferromagnetic behavior of ZnNiO was observed on increasing the Ni concentrations beyond 15% due to anti-ferromagnetic (AFM) ordering of Ni^2+^ ions [17,31].

On a similar note, Ni doping also brings significant changes in the ZnO’s crystal symmetry, local chemical environments and charge transfer mechanisms in addition to disorder and local polarizability, which are correspondingly proved from the XPS studies and the Raman scattering analysis [32,33]. Russo et al., using Raman analysis, successfully predicted presence of local structural strain in doped ZnO nanoparticles [34]. Similarly, oxidation states of the as-incorporated Ni atom in ZnO host affect carrier density, mobility and opto-spintronic transport processes. [35,36]. The XPS studies show that Ni^2+^ ion replaces Zn^2+^ ion in the ZnO cationic sub-lattice [35,37]. Ni ions’ incorporation in ZnO nanoparticles can produce large amounts of surface defects on the nanoparticle surface resulting in the band gap tuning [33]. Fabbiyola et al. found that ≈5–20% Ni concentration reduces ZnO nanoparticles’ size, causing a blue shift in the energy gap observed using optical absorption derived from the Kubelka-Mulk’s function of DRS data [19].

Nevertheless, the functional aspects of doped ZnO ternaries, especially optoelectronic and spintronic properties, depend not only on the type of synthesis method used but also on level of control over dopant substitution in host-lattice [20,35,37,38,39]. It is very important to check the oxidation states of the dopants incorporated in the host lattice to study the doping effects on the carrier density, mobility and other transport and optical properties [40]. Oxidation states of the dopant may vary depending on precursor composition, synthesis method, and annealing treatments [37]. Consequently, different synthesis methods may give contradictory trends of free carrier concentration. Despite many reports being available on TM atom doped ZnO powders, most of them fail to properly describe the effects of dopant oxidation states on optical and structural changes [41]. Here, we attempt to investigate the effects of Ni doping on optical and structural properties of ZnNiO nanoparticles.

In this report, we present a simpler method of ZnNiO nanoparticles synthesis using a single pot sol–gel technique. Our choice of the Ni doping range is based on an observable optical absorption shift owing to expected doping induced band structure modifications. It has been reported that significant optical and structural variations are possible in ZnNiO nano-powders prepared by solid-state reaction technique at 0%, 3%, 5%, 7% and 10% Ni doping levels [29]. Similarly, changes in optical processes in ZnNiO nanoparticles have also been reported for Ni concentration in the range of 5%–20% [19]. In this report, effects of dopant substitution are studied in respect to variations of structural, morphological and optical traits of ZnNiO nanoparticles. Any enhancement in the UV-blue emission from ZnNiO nanoparticles can bring a new functionality among the ZnO-based DMS materials. It would be interesting to see the effects of Ni doping on the wurtzite symmetry of ZnNiO nanoparticles or presence of any doping induced crystalline phase transformations. Moreover, the knowledge of chemical states of dopant in the host lattice would be imperative in determining the mechanism of optical processes such as Burstein-Moss shift vis-à-vis Ni doping percentage.

## 2. Experimental Details

### 2.1. Synthesis of ZnNiO Nanoparticles

ZnNiO nanoparticles are prepared by single-pot sol–gel method, in which precursors like Nickel (II) nitrate hexahydrate (Sigma-Aldrich, USA, 99.5% purity) and Zinc (II) acetate dihydrate (Sigma-Aldrich, USA, 99.8% purity) are mixed in assorted molar ratios of Ni source, 1%, 3%, 7% and 10%. Isopropyl alcohol (Sigma-Aldrich, 99.5% purity), is used as a solvent in the bath reactor. An aqueous stock solution of zinc precursor (0.5 M), 3.5 g of Zn(II)·(CH_3_COO)_2_·2H_2_O powder, is first dissolved in 32 mL of isopropyl alcohol to initiate the hydrolysis. Then assorted weights, 0.047 g, 0.14 g, 0.33 g and 0.47 g of Ni precursor, Ni(II)·(NO_3_).6H_2_O, are correspondingly added to the respective Zn stock solutions and the mixtures are stirred for 10 min at room temperature. Furthermore, a suitable rate of hydrolysis is achieved by alkalization with drop-wisely addition of 1.3 mL of mono ethanolamine (MEA) in the solution. Throughout the ZnNiO synthesis, the molar ratio between isopropyl alcohol, zinc precursor and MEA is kept constant ≈200:10:1. Afterwards, the temperature of the solution is raised to 60 °C with continuous stirring for 2 h to complete the alcoholic condensation reaction, and finally a gel is obtained in the form of a fine white colored powder. The obtained gel is dried for 1 h at 250 °C temperature in the oven leading to water and alcohol evaporation and subsequently calcined in air at 600 °C for 5 h. For the assorted weight percentages of Ni, 1% to 10%, different colors of ZnNiO powders are obtained ranging from off-white to grayish-white with respect to increasing Ni concentration.

### 2.2. Characterization of ZnNiO Nanoparticles

XRD technique is employed for structure examination of as-prepared ZnNiO in powder form. Powder X-ray diffractometer (Ultima-4, Rigaku, Japan) is used for XRD analysis of powdered samples. XRD measurements are obtained in a 2θ range from 20°–80° using CuK_α_ (λ = 1.5406 Å) radiation source. Evaluation of microstructure and morphology of ZnNiO powders is performed using high-resolution scanning electron microscope (JSM-7600F, Jeol, Japan) and recorded at 100,000× and 25,000× scan magnifications. XPS technique is used to quantitatively probe chemical species and oxidation states. Mg-kα radiation (1253.6 eV) radiation source is used in XPS instrument (ESCA-II, Omicron, Germany) to record spectra at 0.05 eV spectral resolution and at 50-μm X-ray beam spot-size. Raman Spectroscopy, a useful method for understanding lattice vibration energies, is used to study characteristic and disorder phonons present in nanoparticles. Raman spectra are recorded in confocal Raman microscope (Horiba spectrometer, Labram HR Evolution, France) at ultra-low frequency compatible scans in 50 to 800 cm^−1^ range using the 633 nm He-Ne laser excitation source. Diffusion reflectance spectroscopy is performed in spectral range of 190-1100nm at ±0.5 nm spectral accuracy using the UV-Vis spectrometer (UV1800, Shimadzu, Japan). The DRS data is collected on pelleted ZnNiO powders hydraulically pressed at 3.5-tons pressure. Photoluminescence (PL) measurements are also performed in the Horiba Labram PL spectrophotometer using 325 nm He-Cd laser excitation source.

## 3. Results and Discussion

**Figure 1:**
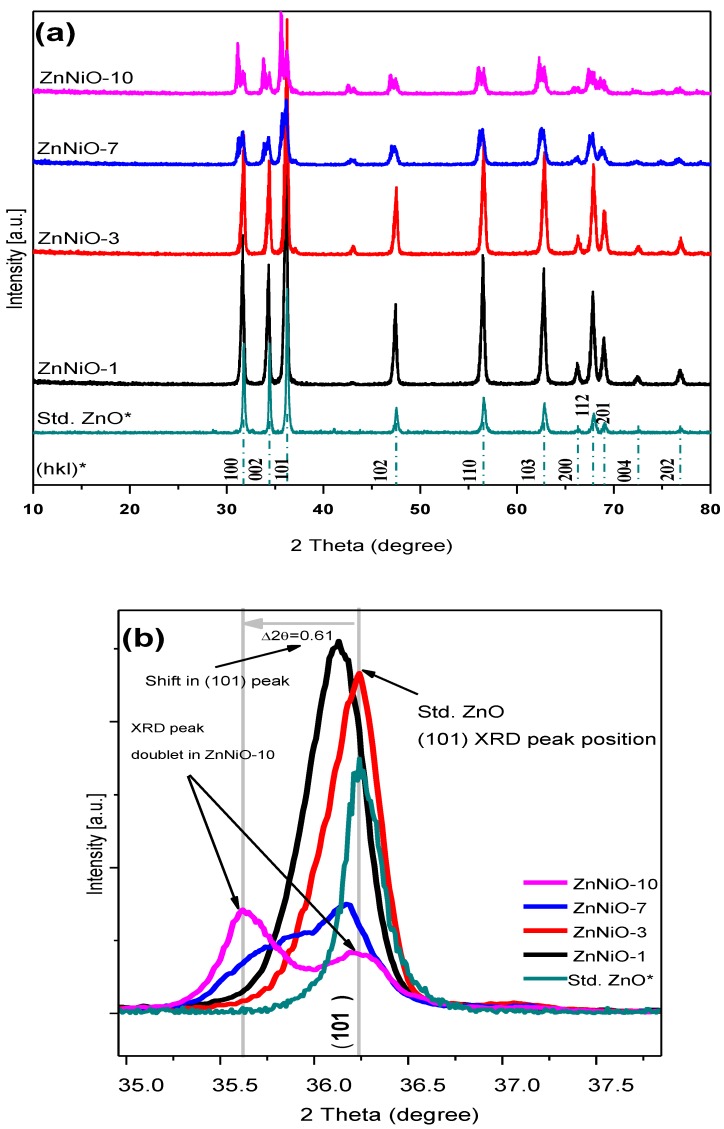
(**a**) XRD patterns of ZnNiO nanoparticles are presented in vertically aligned mode in increasing doping percentages. The Ni doping increases in samples ZnNiO-1, -3, -7 and -10. Asterix sign (*) shows the standard ZnO’s XRD pattern and its (hkl) reflection positions as adopted from the XRD card, RRUFFID-R050492. (**b**) shows (101) -peak profiles of most intense XRD reflection in ZnNiO indicating a 2θ shift upon doping and a gradual development of 101-doublet peaks.

XRD patterns of ZnNiO powders are shown in Figure 1a. For the sake of clarity, a vertical offset of XRD patterns with Ni doping is shown in the figure. All samples exhibit characteristic ZnO peaks at (100), (200) and (101) XRD reflections confirming the wurtzite structure of ZnNiO. Additionally, the sharper and more intense nature of XRD peaks at lower Ni concentrations indicates a strong wurtzite character of ZnNiO nanoparticles. However, on further doping, the XRD peaks become broader and less intense, indicating decay in the wurtzite character. Nonetheless, the wurtzite structure remains a dominant phase, during compositional variation of ZnNiO nanoparticles, indicating a substitutional solid solution formation. Furthermore, absence of any new XRD peaks on Ni doping implies absence of secondary structural phases. Nevertheless, upon careful examination of the XRD patterns, the XRD peaks are found to be dividing into a doublet peak structure; a smaller XRD peak originates nearby each major XRD peak, reflecting a systematic development of structural changes in ZnNiO at higher Ni doping. The observation is further clarified from the XRD peak profiles of 101-reflections plotted in Figure 1b, which clearly show shifted and less intense peaks, reflecting a decay in the wurtzite nature of ZnNiO. The (101)-peak becomes a clear doublet peak at 10% Ni content, Δ2θ ≈ 0.61 for ZnNiO-10, apparently indicating reduction in wurtzite symmetry or a weaker lattice strain induced zinc-blende symmetry at higher doping. The nano phase ZnNiO has already been reported to grow in both wurtzite and zinc-blende metastable phases owing to similarity in their anionic coordination [17]. During the doublet development on doping, as shown in Figure 1b, the lower peak at 2θ ≈ 35.5° can best be attributed to a weaker zinc-Blend symmetry of ZnNiO nanoparticles. Herewith, we reject the possibility of any secondary oxide phase formation on doping in the light of absence of NiO’s XRD peaks. It should be noted that NiO crystals typically adopt hexoctahedral cubic system (XRD card number: RRUFFID-R080121 [42]) and a major XRD reflection of NiO (111) is positioned at 2θ ≈ 37.45. The XRD peaks related to NiO cubic phase are completely absent in the ZnNiO XRD patterns at all doping levels implying absence of new phases. Moreover, the XRD peak shift value of Δ2θ in the present studies can be best ascribed either to uniform crystalline strains present in ZnNiO lattice as a result of ionic radii mismatch between Zn^2+^ (0.74 Å) and Ni^2+^ ions (0.69 Å) or to reduced symmetry defects in the crystal structure [20]. The results of the observed crystalline strain behavior in ZnNiO nanocrystals are also summarized in Table 1. Typically, the strain increases in ZnNiO nanoparticles with increasing Ni, and its behavior agrees very well with the increase in unit cell volume.

Crystallite size (D) for nanoparticles in spherical approximation can be easily calculated by measuring the (101)-peak broadening (β) and applying Scherrer’s formula, D=K/β, where constant *K* depends on the x-ray source and θ-position of the peak used [43]. The Scherrer’s particle sizes for ZnNiO nanoparticles are summarized in Table 1. ZnNiO particle sizes were found to systematically decrease from 32 to 14 nm with increasing Ni concentration, as shown in the table. The lower nanoparticle size at higher Ni doping also indicates higher doping related defects affecting the growth of crystallites. Lattice parameters and unit cell volume of ZnNiO were also found to be increasing with Ni content in nanoparticles, as shown in Table 1. Change in the unit cell volume can also be ascribed to the presence of uniform crystalline strain and difference in the ionic radii of the substituted Ni ions in comparison to Zn^2+^ cations [43].

**Figure 2:**
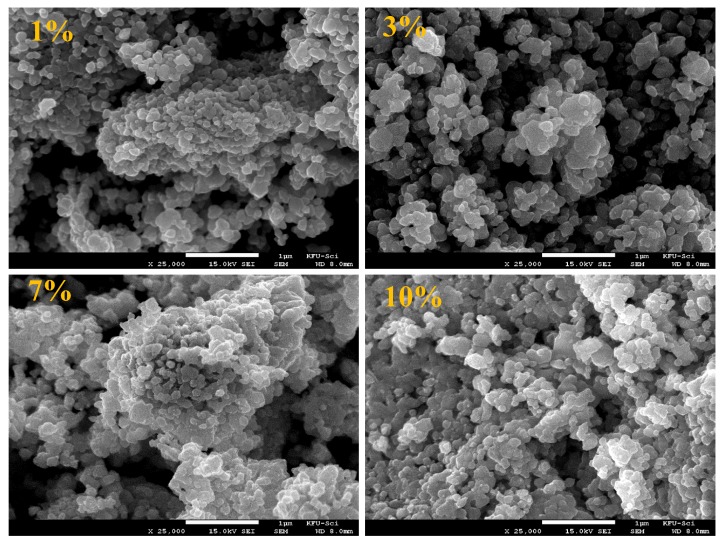
SEM images recorded at 25,000× magnification showing surface morphology of ZnNiO powders at 1%, 3%, 7% and 10% Ni doping percentages. The white colored bar-scale in the bottom of each image represents 1 μm.

**Figure 3:**
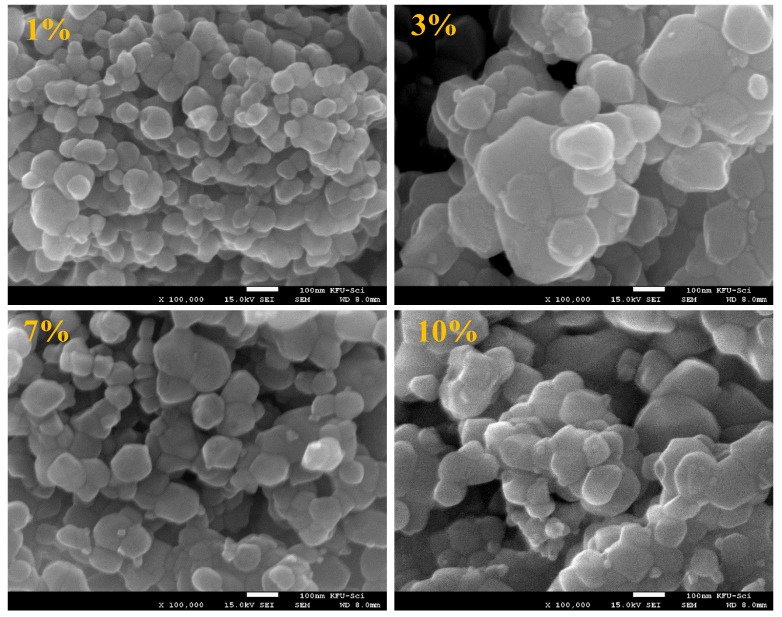
SEM images recorded at higher magnifications (100,000×) showing the granular morphology of ZnNiO powders at 1%, 3%, 7% and 10% doping percentages. The white colored bar-scale in the bottom of each image represents 100 nm.

Electron microscopic images of ZnNiO nanoparticles with different Ni ion concentrations are shown in Figure 2 and Figure 3 at 25,000× and 100,000× magnifications, respectively. In the wider area scan of Figure 2, a surface morphology consisting of nanoparticles is distinctively apparent within the rounded shaped regions of larger agglomerations. Moreover, uniformly distributed nanoparticulate morphology is also obvious among all the sol–gel prepared ZnNiO powders. The clusters are characteristically formed in comparable sizes and shapes, which confirms a uniform sol–gel procedure is adopted during the ZnNiO syntheses. SEM images for ZnNiO samples with higher magnification scanning are shown in Figure 3, depicting a granular morphology of nanoparticles. The grains are seemingly hexagonally faceted, indicating conservation of wurtzite structure of ZnNiO during Ni doping. Sharply hexagonally faceted grains can even be observed for nanoparticles at 3% and 7% Ni doping percentages. The higher magnification electron microscopy confirms the identical nature of as-formed grain sizes, along with uniform size distributions among all samples. Comparatively higher porosity and intragranular spacing is observed in the granular morphology of ZnNiO with lower Ni content—1%. On increasing Ni content in remaining ZnNiO samples, the particle sizes remain constant at ≈52 nm; nonetheless, the morphological compactness increases along with reduction in the intragranular voids.

**Figure 4:**
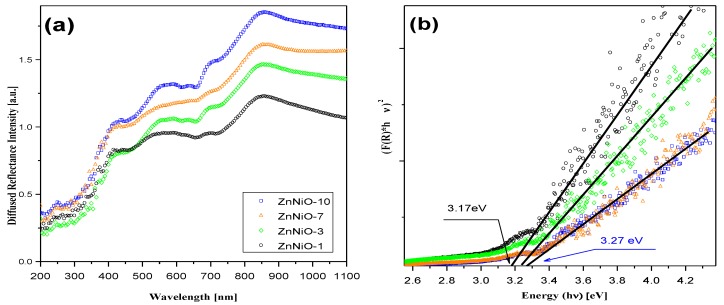
(**a**) The UV-VIS diffuse reflectance spectra for ZnNiO pellets are vertically stacked and plotted in the increasing Ni doping order. The doping percentages in samples, ZnNiO-1, -3, -7 and -10%, are correspondingly plotted in black, green, red and blue colored spectral lines. (**b**) Kubelka-Munk fittings of the DRS are plotted in the right-hand side figure.

Optical absorption properties and electronic bandgap are determined using a straight-line fit on Kubelka-Munk function calculated from the diffused reflectance data. The DRS data, as shown in Figure 4a, is plotted in the UV-Vis range for ZnNiO nanoparticles with vertically decreasing Ni concentration. The DR values recorded at different compositions of ZnNiO show a rapid decrease at wavelengths lower than 400 nm. The observed reflectance feature is the most common feature among ZnNiO nanoparticles in UV light range due to fundamental absorption onset involving opto-electronic transition from valance band (VB) to conduction band (CB) [1,44]. In general, the optical reflectivity gradually decreases with Ni doping owing to an upsurge in the dopant related internal light scattering events [19]. Indeed, the Ni doping-induced increase in bond-polarizability in ZnNiO can also lead to higher internal light scattering and low DRS intensity [45,46].

The Kubelka-Munk (KM) function, $F\left(R\right)=\frac{K}{S}={\left(1-R\right)}^{2}/2R$; where *K*, S and *R* denote absorption, scattering and reflection coefficients, respectively, is a direct measure of the absorbance from the sample’s reflectivity data. The KM function of the diffused reflection spectrum can be used in the calculation of electronic bandgap using the onset of the fundamental absorption edge [47]. The function [(F(R)×hν]^2^, which is a direct measure of the absorbance in the material, is plotted with respect to the photon energy (hν, eV) in Figure 4b. A straight-line fit is performed in a linear region of the KM function and extrapolated to baseline absorbance on the hν-axis to find the Tauc’s optical bandgap, as shown in the figure. A slightly blue-shifted bandgap (Δ ≈ 0.1 eV) is observed in ZnNiO-7 and -10 samples in comparison to the ZnNiO-1 and -3 samples. The bandgaps in the latter two samples with lower Ni concentrations are closer to the reported band gap (~3.1 eV) of standard ZnO [20]. A small blue shift of ~0.1 eV observed with Ni doping is understandably not related to quantum size confinement effects owing to non-variance of ZnNiO particle sizes during Ni doping. Fabbiyola et al., have attributed enhancements in the KM function in ZnNiO to Ni doping due to the formation of surface states which significantly increase the optical absorption [19]. However, we believe the increased *F*(*R*) in this study is due to doping induced Burstein-Moss effect. In comparison to bivalent dopant (Ni^2+^) substitution, the trivalent dopant (Ni^3+^) substitution in the ZnO lattice replacing the divalent Zn^2+^ has been reported to provide an additional electron for conduction [47]. The Ni atom donating electrons to the conduction band suggestively increase the Fermi level and itinerant related absorption toward the higher energy [48]. Moreover, the KM function value in the UV-blue region is typically higher for ZnNiO with higher Ni doping, and that also indicates an increase in the overall number of optical absorption processes. Furthermore, the Ni doping-related increase in ZnNiO bandgap has also been ascribed to increase in excitonic density and excitonic mediated optical transitions resulting in highly diffused nature of DRS [49].

**Figure 5:**
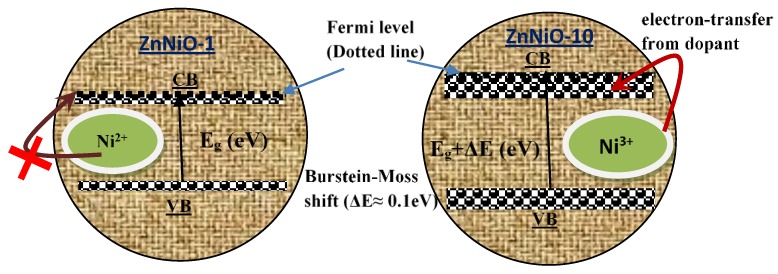
Optical transition processes taking place differently in ZnNiO nanoparticles with l% and 10% Ni doping levels. Higher Kubelka-Munk function values F(R) are expected in UV-blue region for the optical transition shown in right-side schematic of the figure.

A possible mechanism of the Burstein-Moss-mediated optical absorption process is depicted in Figure 5 (right), illustrating itinerant related optical absorption and electron transfer from the trivalent dopant (Ni^3+^) to the conduction band (CB) resulting in upshifting of the Fermi-level. Our XPS studies, which are discussed later in the manuscript, also indicate presence of trivalent Ni doping. Thus, the trivalent substitution of Ni atom in the inherently divalent cationic sub-lattice of Zn^2+^ ions could give electron donating character to nickel atom resulting in the higher carrier concentration effectively lifting the Fermi energy position in the band structure. While the electron transfer is proscribed in the divalent Ni substitution, as shown in Figure 5 (left).

**Figure 6:**
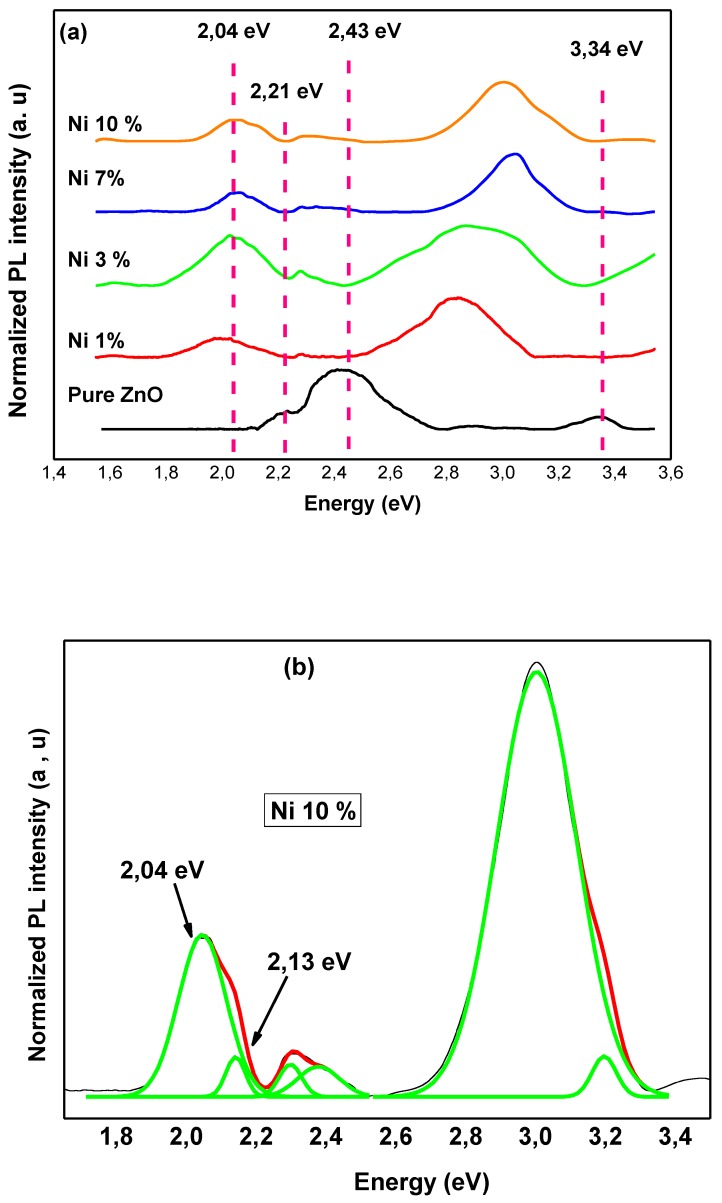

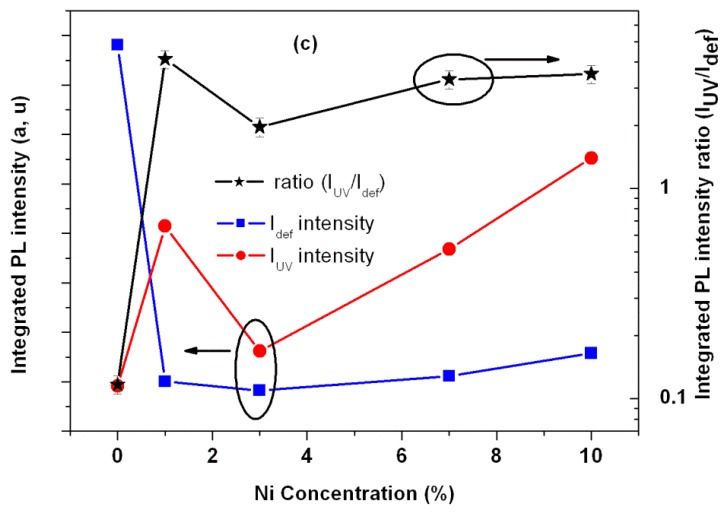
(**a**) Room temperature PL spectra of ZnNiO nanoparticles. (**b**) PL spectra of 10% ZnNiO along with the Gaussian fit. Luminescence arising from the pure bulk ZnO is shown as a reference. (**c**) Integrated PL intensity of the UV band (I_UV_), the wide defect related band (I_def_) and relative PL peak intensities ratio R (I_UV_/I_def_) vs Ni concentration.

To study the intrinsic and extrinsic defects, photoluminescence (PL) spectroscopy was carried out. Figure 6 shows room temperature PL spectra of ZnNiO nanoparticles excited at 325 nm. It can be seen from the pure ZnO (reference sample) that the PL spectra are mainly comprised of UV emission peaks at 3.34 eV (370 nm) followed by a high-intensity broad visible band with multiple sub-peaks in the range of 2.1–2.75 eV (450–590 nm). The UV emission is attributed to the recombination of free excitons in the near band edge (NBE) of ZnO, whereas the visible band is generally interpreted as the reason for the defects [8,50,51,52,53]. Meanwhile, the origins of different visible emissions are still not fully assumed and different controversial hypotheses have been offered [8,50,51,52,53]. In our case, the emission features at 2.43 eV (510 nm) and 2.21 eV (560 nm) are related to antisite oxide (OZn) and to transmission from conduction band to vacancy, respectively.

It is shown in Figure 6a that with Ni incorporation, the UV emission intensity typically increases and is shifted to the higher energy, while the intensities of the visible peaks decreases. The results show that Ni doping increases the electron concentration, and that there is a concomitant decrease in the OZn and vacancy defects. On the other hand, additional broad band emissions start becoming apparent at low energy (1.8–2.2 eV) in ZnNiO samples. The broad band is deconvoluted for two different peaks, as shown in Figure 6b. The emission peak at 2.04 eV (607 nm) corresponds to Ni-related defects, and the peaks at 2.13 eV (582 nm) correspond to donor–acceptor pair recombination [52]. For more details, Figure 6c shows changes in the integrated PL intensity of the UV band (I_UV_), the wide defect-related band (I_def_) and the relative PL peak intensities ratio R (I_UV_/I_def_). From our PL observation, it is evident that Ni doping results in enhancement of luminescence arising in the UV band. Meanwhile, the wide defect-related band decreases in comparison to that of the pure ZnO luminescence, as observed in the same plot. Furthermore, the intensity ratio R increases with increasing Ni content and gets saturated at 7% and 10% Ni concentration, showing higher enhancement of UV emission. It is interesting to note that the UV emission is also blue-shifted when the Ni concentration increases from 1% to 10% due to the Burstein-Moss effect [8]. The electron-doped ZnNiO with a high concentration of n-type states populated within the conduction band moves the Fermi level to higher energies. The filling of the conduction band by electrons will generally result in a blue shift [8].

**Figure 7:**
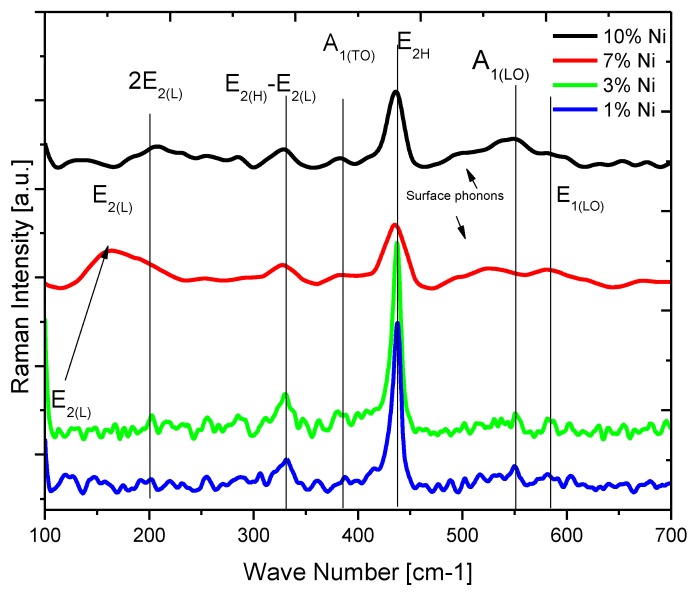
Room temperature micro Raman spectra for ZnNiO nanoparticles recorded in the confocal mode. Vertical lines show the positions of different normal modes of Raman vibrations. Ni content in the ZnNiO samples are written by Ni-1%, -3%, -7% and -10%, respectively, and the corresponding Raman spectra are plotted in blue, green, red and black colors.

Raman analysis is very useful for finding Ni^2+^ doping-related changes in ZnNiO crystalline structure, such as the formation of structural disorders, presence of vacancies, or defects in host lattice [32,33]. The host ZnO has a wurtzite structure belonging to P63mc space group symmetry [54]. Eight active normal vibrational modes are present in the wurtzite ZnO comprising of two acoustic modes and six optical modes, of which four optical modes are Raman active [55]. Before explaining the Raman spectra of ZnNiO, a brief description of expected vibrational modes is required. The major Raman modes of ZnO, in Group theory’s Mulliken notations, are A_1_, 2E_2_ and E_1_. The A_1_ mode is symmetric to principal rotation axis (c-axis) and shows a mode split in the form of A_1_(TO) and A_1_(LO) modes relative to transverse and longitudinal one-dimensional atomic vibration. The split is due to the macroscopic electric field of LO vibration increasing the restoring force and stiffness of the bond, thus increasing the split A_1_ mode’s energy. Similarly, the two-dimensional E_1_ mode is perpendicular to the c-axis, with displacements of the O- and Zn sub-lattices also showing polarity-induced stiffness and manifesting in the E_1_ mode’s LO/TO splitting. However, the E_2_ mode is asymmetric on the c-axis and the polarity-induced macroscopic field can be easily cancelled out due to asymmetric motion of the sub-lattices. Hence, the LO/TO splits do not take place and the mode remains non-polar. Nevertheless, the E_2_ mode further shows two components, low (E_2L_) and high (E_2H_) energy modes due to atomic mass difference between the Zn and O atoms. Additionally, some higher-order difference modes and surface phonon modes are also expected in wurtzite ZnO. The usual Raman modes are often reported in the literature for the wurtzite phase of ZnO: E_2L_-E_2H,_ A_1_(TO), E_2H_, surface phonons and A_1_(LO) [33,34]. Raman spectra recorded for ZnNiO samples are shown in Figure 7 and all the usual Raman peaks are appropriately marked. The positions of the marked Raman peaks in ZnNiO are generally in agreement with the available literature [30,33,34]. The presence of all the expected Raman modes in the figure indicates conservation of wurtzite phase in ZnNiO.

The highest intensity E_2H_ mode, related to two-dimensional doubly degenerate vibrations of O-atoms, is very sensitive to internal stresses [34]. Ni doping has been reported to introduce new stresses and disorder in the cationic sub-lattice reducing the overall wurtzite symmetry of ZnNiO [30,33]. Ni doping can also distort the local charge distributions in cationic sub-lattice leading to reduction in bond polarizability and reduced Raman intensity. E_2H_ mode intensity in ZnNiO is found to be reduced by ≈50% at 7% and 10% Ni content in comparison to other compositions. The reduced Raman intensity indicates introduction of internal stresses in material as a result of doping. Both the Raman analysis and the XRD structural analysis point towards the presence of higher stress in ZnNiO samples. Moreover, doping-induced stress is also expected in ZnNiO samples because of the possibility of differences in dopants’ radii of Ni^2+^ and Ni^3+^ ions [56].

The Raman modes, A_1_(TO) and A_1_(LO), are positioned at 380 and 550 cm^−1^, respectively, and according to the Lyddane–Sachs–Teller (LST) rule the mode positions indicate the ionic character of ZnNiO [34,57]. The LST rule explains that the square of the ratio of LO and TO frequencies in the ionic lattice should be equal to ratio of static (8.5 for ZnO) to optical (4.1 for ZnO) dielectric constants. The A_1_(TO) and A_1_(LO) modes are also found to shift to higher frequencies with increasing Ni content in ZnO samples. The modes involve symmetric vibration of both Zn and O sub-lattices, and they are sensitive to the presence of Ni atoms in Zn sub-lattice. The ionic radii of Ni ions in octahedron coordination are 0.69 Å, 0.56 Å, and 0.48 Å for Ni^2+^, Ni^3+^ and Ni^4+^ ions, which are lower in size than Zn^2+^, 0.74 Å. Low ionic radii cause a reduced distance in between the two sub-lattices and that increases the force constant of bond and vibration frequency. The intensity of A_1_(LO) mode was found to increase with Ni doping, which can be ascribed to the involvement of O-atoms only in the vibration, while the presence of vacancies can increase the mode intensity due to the change in bond polarizability.

The E_2_ overtone and difference modes arise at different positions due to higher-order 2E_2L_ and difference E_2H_-E_2L_ modes at peak positions 200 cm^−1^ and 330 cm^−1^, respectively. Disorder modes are also present in the nanoparticles, as is clear from Figure 7, at 530 cm^−1^, indicating the presence of either higher surface disorder, defect density or non-uniform strain, especially at 7% and 10% Ni doping percentages. The disorder modes have often been described as originating from the doping related structural defects and that results in manifestation of several broader line width phonons in doped ZnO [34].

**Figure 8:**
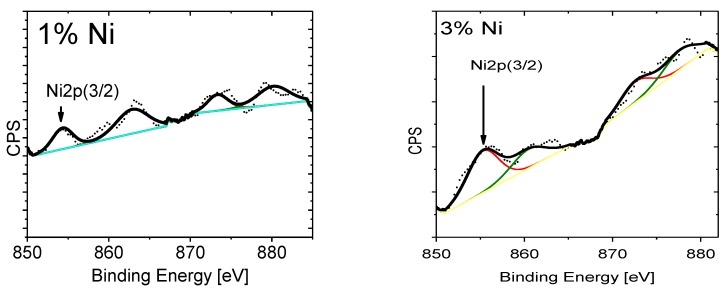

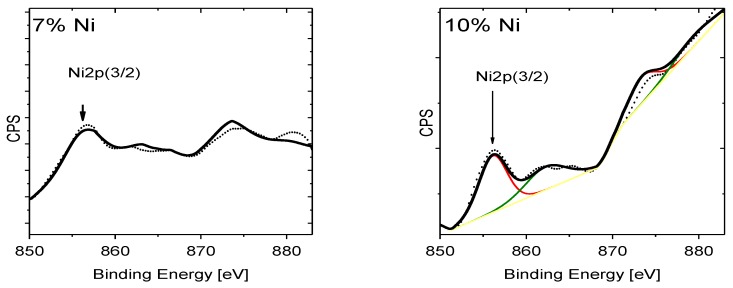
High-resolution XPS spectra are plotted for the Ni_2p_ photoelectrons. XPS is recorded for the ZnNiO samples containing 1%, 3%, 7% and 10% Ni weight percentages.

**Figure 9:**
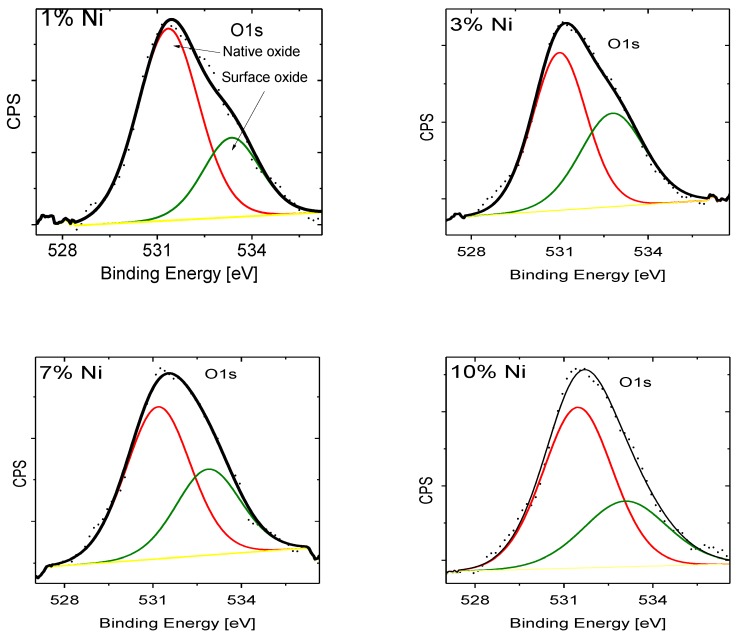
XPS recorded for the ZnNiO samples containing 1%, 3%, 7% and 10% Ni weight percentages. High-resolution XPS spectra are plotted for the O_1s_ photoelectrons.

**Figure 10:**
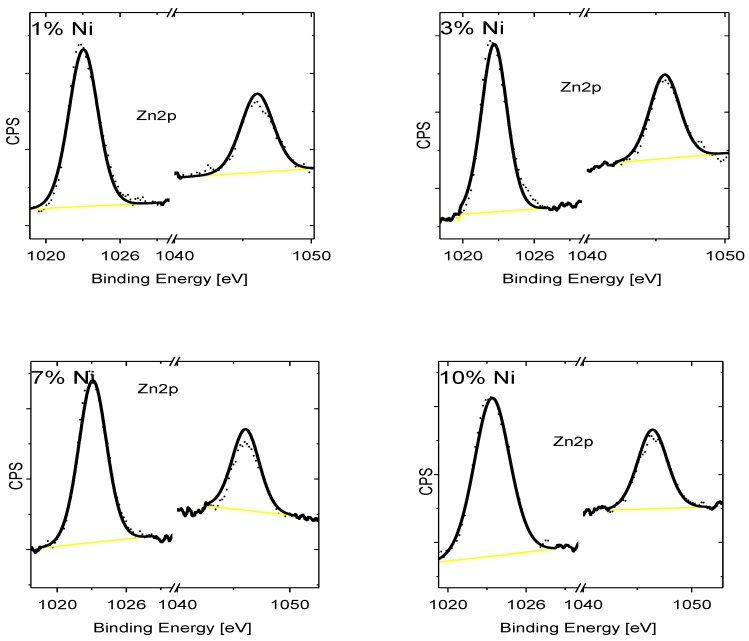
XPS recorded for the ZnNiO samples containing 1%, 3%, 7% and 10% Ni weight percentages. High-resolution XPS spectra are plotted for the Zn_2p_ photoelectrons.

XPS spectra for Ni_2p_, O_1s_ and Zn_2p_ photoelectron binding energies (BE) in ZnNiO nanoparticles are shown in Figure 8, Figure 9 and Figure 10, respectively, for assorted Ni doping concentrations. The Ni_2p_ spectrum shows a general feature of two distinct XPS peaks at BE values of 854.3 eV and 873.3 eV for Ni^2+^ oxidation state and correspondingly related to 3/2 and 1/2 spin-split components of Ni_2p_ electrons. Excluding the ZnNiO with 1% doping level, the Ni_2p_ XPS peaks, as shown in Figure 8, exhibit a general feature of blue shifted binding energy and XPS peak broadening. Typically, the BE shift increases with doping of ZnNiO, and the BE energy is blue shifted to 856.1 eV at the 10% Ni doping level, indicating the presence of both Ni^2+^ and Ni^3+^ oxidation states. A difference of ≈2.7 eV has been reported previously in between the Ni^2+^ and Ni^3+^ oxidation states [58]. The Ni_2p_ BE blue shift (−1.8 eV) indicates the presence of the additional Ni^3+^ oxidation state. Thus, the Ni_2p_ XPS results directly point towards substitution of multivalent Ni dopants, both Ni^2+^ as well as Ni^3+^, in ZnNiO nanoparticles, especially in the ZnNiO-10 sample. Conversely, in the 1% Ni doped sample, ZnNiO-1, should only have the divalent Ni doping because of the absence of the BE shift. The aforementioned Burstein-Moss blue shifts can also be easily explained by using the observation of Ni^3+^ oxidation states in the ZnNiO-10 sample; the Ni atom is indeed behaving as a donor atom in ZnNiO nanoparticles at higher doping percentages ≥7%.

A deconvolution of the XPS peak of O_1s_ electrons in ZnNiO nanoparticles shows two peaks; the first peak is indicative of the native oxygen, and the second deconvoluted peak positioned at 532.5 eV is related to the oxygen defects [58]. Indeed, the oxygen defects are found to increase at higher Ni doping, since the second O_1s_ peak, as shown in Figure 9, becomes larger in area with doping, and that is also indicative of the formation of more oxygen defects due to Ni^3+^ substitution in the Zn^2+^ cationic sub-lattice. At higher Ni doping, the XPS spectra of the Zn_2p_ binding energy, see Figure 10, show relatively little change in the positions 1023 eV and 1046 eV of both types of 2p electrons of Zn ion. The Zn^2+^ oxidation state remains invariant to the doping.

## 4. Conclusions

A simple sol–gel method is used to prepare ZnNiO nanoparticles at assorted doping levels by increasing the Ni concentration to 10%. The dopant concentration was clearly found to significantly affect optical and structural properties of ZnNiO nanoparticles. XRD results indicate a substitutional solid solution phase among the ternary nanoparticles. No impurity phase is detected at all the assorted Ni doping percentages and all the observed XRD reflections can only be ascribed to wurtzite structural phase of ZnNiO. However, reduced structural symmetry, crystal strain and structural defects are detected during the XRD analysis of ZnNiO nanoparticles. XRD peak-shifting and -splitting become clear at higher Ni doping levels >7% and are attributed to the presence of crystal strain. Furthermore, the doping related decrease in Scherrer’s particle size from 32 nm to 14 nm is also noted in ZnNiO nanoparticles. Spherical nanoparticle morphology is observed in SEM imaging with larger agglomerations of ZnNiO nanoparticles (≈52 nm) forming bigger clusters at higher doping. Kubelka-Munk function F(R) calculated from the DRS data is used to find the fundamental optical absorption onset. A small Burstein-Moss shift ≈0.1eV is observed in the linear-fit of KM function, which is credited to electrons transferred from the Ni dopant to the ZnNiO conduction band. Similarly, PL studies reveal intense UV luminescence in ZnNiO nanoparticles in comparison to pure ZnO, and the UV PL emission peak is also found to be blue-shifted ~0.15 eV with 10% Ni doping, further corroborating the Burstein-Moss effect. Raman analyses show a complete agreement with XRD structural studies. Doping-induced reduction in the Raman peak intensity of E-mode indicates a gradual decay of wurtzite character of ZnNiO nanoparticles. Additionally, polar Raman modes exhibit Ni doping related increased bond stiffness indicating presence of crystal strain in ZnNiO nanoparticles. Several disorder Raman modes are also observed in ZnNiO nanoparticles, confirming the presence of structural defects. Lastly, XPS studies show the presence of both Ni^2+^ and Ni^3+^ oxidation states at higher Ni doping. In this report, we have unequivocally shown the importance of multivalent Ni dopant states in the observed optical and structural changes of ZnNiO nanoparticles.

## Figures and Tables

**Table 1:** Summary of structural properties present in the assorted samples ZnNiO powders calculated by XRD data fitting. Columns from left represent Ni content (weight percentage), average crystallite size (D) in nanometers (nm), lattice constants (a, c) and their ratio (c/a), bulk strain (Δd/d) present in ZnNiO nanoparticles and their unit cell volume (V).

Doping-Percent	D, nm	a, Å	c, Å	c/a	Strain	V, Å^3^
1%	29.8 ± 1.57	3.23 ± 0.01	5.22 ± 0.01	1.600 ± 0.01	0.19	47.99
3%	32.2 ± 2.41	3.23 ± 0.01	5.21 ± 0.01	1.597 ± 0.01	0.01	47.88
7%	18.1 ± 1.37	3.28 ± 0.02	5.22 ± 0.02	1.594 ± 0.01	0.36	48.57
10%	14.1 ± 0.80	3.30 ± 0.02	5.24 ± 0.04	1.586 ± 0.02	0.68	49.54
